# Child malnutrition in Afghanistan amid a deepening humanitarian crisis

**DOI:** 10.1093/inthealth/ihac055

**Published:** 2022-09-05

**Authors:** Zainab Syyeda Rahmat, Hania Mansoor Rafi, Arsalan Nadeem, Yumna Salman, Faisal A Nawaz, Mohammad Yasir Essar

**Affiliations:** Faculty of Medicine, Dow Medical College, Dow University of Health Sciences, Karachi, Pakistan; Faculty of Medicine, Dow Medical College, Dow University of Health Sciences, Karachi, Pakistan; Faculty of Medicine, Allama Iqbal Medical College, Lahore, Pakistan; Faculty of Medicine, Dow Medical College, Dow University of Health Sciences, Karachi, Pakistan; College of Medicine, Mohammed Bin Rashid University of Medicine and Health Sciences, Dubai, United Arab Emirates; Kabul University of Medical Sciences, Kabul, Afghanistan

**Keywords:** Afghanistan, child malnutrition, humanitarian crisis

## Abstract

Decades of political turmoil and stifling war, among other issues, has turned Afghanistan into the world's largest humanitarian crisis. Just 4 months after the Taliban seized control, the majority of the Afghan population face starvation, with >3.3 million children unable to afford basic food resources, leading to dozens of deaths every week. Restrictions on humanitarian assistance, withholding of vital food supplies and inadequate medical care play a major role in exacerbating the rates of malnutrition in the vulnerable paediatric population. Global interference is warranted to avoid unfathomable consequences in mitigating this public health catastrophe.

## Introduction

Twenty years of war in Afghanistan has cost >1.5 million lives.^1^ However, if the current deepening humanitarian crisis in Afghanistan is not adequately addressed, more people will die of starvation than in the past 20 y of war.^2^ The history of political unrest along with inadequate economic opportunities has severely diminished the purchasing power of the public. With a population of >40 million in 2022, Afghanistan has witnessed a sharp increase in poverty, with levels increasing from <40% in 2007 to almost 60% in 2016.^3,4^ By the end of 2021, half of the population was living below the poverty line.^5^ Monetary poverty is higher among children than adults, with the highest levels reported in the youngest age groups. About 58% of children <17 y of age are impoverished, with the number increasing to 61% of children <9 y of age.^6^ In general, poverty rates are highest in rural areas, which is home to 83% of the population. The poverty status is lowest in Kabul, affecting 12% of the population.^7^ A similar trend is noted in the levels of food insecurity, with 30% of Afghans affected in 2011–2012 and 45% affected in 2016–2017.^4^ A worsening humanitarian crisis is demonstrated by these trends and has resulted in the malnourishment of millions of children and adults in Afghanistan.^4^

There are currently 12.9 million children in Afghanistan and >1200 births are reported each year.^8,9^ The percentage of children <5 y of age with registered births is at a mere 51%, indicating difficulty in acquiring accurate statistics for children <5 y of age.^3^ Regardless, the number of children <5 y of age being admitted to the hospital with severe acute malnutrition (also known as wasting) has risen significantly from 16 000 admissions in 2020 to 28 000 in 2022.^5^ This increase is attributed to the recent political turmoil of 2021, with many international sanctions and withdrawal of foreign aid causing the collapse of the Afghan economy, pushing thousands into poverty.^5^

Malnutrition is defined as deprivation of vital vitamins, minerals and nutrients essential for the normal function of tissues and organs that can be a result of either undernourishment or overnourishment.^10^ Children are more susceptible to diseases and mortality because of undernutrition. About 45% of deaths among children <5 y of age in lower- and middle-income countries have links to undernutrition.^11^ The most important factors that cause child malnutrition are unsanitary household environments, food insecurity and lack proper healthcare, according to the United Nations Children's Fund (UNICEF).^12^ Acute malnutrition has been one of the health sectors’ key concerns in Afghanistan, as the nation is one of the most resource-deprived countries in South Asia.^5,6^

In 2021, 14 million Afghans were noted to be deficient in food supplies, with 95% of households not eating enough.^13^ In the same year, the Global Hunger Index ranked Afghanistan at 103rd of 116 nations, indicating a ‘serious’ level of hunger.^14^ It comes as no surprise that the levels of food insecurity have risen due to the recent abrupt halt of foreign aid, as almost 75% of public spending came from foreign aid grants.^15^ In 2021, half of Afghan children <5 y of age were expected to have acute malnutrition and at least 1 million children were expected to die due to severe malnutrition.^13^ In addition, only 12% of Afghan children 6 months–2 y of age receive the appropriate composition of meals in quantities adequate for their age.^16^ The latest statistics reveal the deaths of 13 000 newborns in Afghanistan since January 2022 due to malnutrition and other health-related diseases.^17^

If left unchecked, the anticipated universal poverty of all Afghanis will result in the wasting of 1.1 million children <5 y of age in 2022.^5^ Therefore the objective of this article is to highlight the child malnutrition crisis in Afghanistan and the aggravating factors, as well as to provide scalable recommendations to alleviate this issue.

## Challenges and implications

Afghanistan has one of the highest rates of stunted growth among children <5 y of age and a staggering 41% of children under the range of normal growth parameters.^16^ Currently14 million Afghani children are vulnerable to starvation and dozens of children <5 y of age are losing their lives to starvation every week.^13^

Prior to the Taliban takeover, the country was already in a hunger crisis due to devastating droughts and years of political turmoil.^5^ The effects of the shift in power have pushed millions to the brink of starvation and has worsened the child malnutrition crisis. These are major challenges that must be dealt with to save the lives of millions of children.

The effects of global aid cessation and international sanctions were felt by almost all Afghanis. For >7 y the citizens of Afghanistan have relied heavily on international financial aid for survival.^15^ Due to freezing of financial assets in 2021, the nation is unable to meet its populations’ basic welfare needs.^15^ This year, 22.8 million Afghanis are struggling with food insecurities.^5^ Of those, 8.7 million are expected to enter critical levels of food insecurity, which is double the number last year and marks a record high for Afghanistan.^8^

Another challenge posed by the lack of international aid and sanctions is the near collapse of the healthcare system amid recurrent infections. A recent measles outbreak has pushed the capacity of the few functioning hospitals to their limits.^18^ A study found recurrent infections caused by acute malnutrition and also noted that children who had diarrhoea in the previous 2 weeks were nearly two times more likely to be critically malnourished.^19^ In Afghanistan, respiratory infections were the cause of more than one-quarter of children dying at <5 y of age, with almost all these deaths being due to community-acquired pneumonia.^20^

Afghan children <5 y of age bear the brunt of indirect conflict mortality, with an estimated 246 million children living in areas impacted by armed conflict, corresponding to 1 out of every 10 children globally.^21^ Armed conflict indirectly leads to ill health by the subsequent destruction of healthcare facilities and social services, mass displacement and increased risk for transmission of diseases.^21^

Lack of awareness of hygienic food practices due to high rates of illiteracy are attributed to worsening child malnutrition.^22^ General dietary knowledge and understanding of food management and health is significantly poor.^8,22^ Customary early-age marriages prior to completing higher education is considered to be one of the reasons for this lack of awareness.^8^ Most children do not consume fruits or vegetables, and only half of Afghan newborns are exclusively breastfed for the first 6 months of their lives, putting them in danger of life-threatening infections.^16^ In 2019 it was reported that for every 1000 babies born in Afghanistan, 60 babies do not make it to their fifth year of life.^23^

Recurrent natural catastrophes have a significant impact on food insecurity, leading to subsequent exacerbation of child malnutrition.^24^ Several droughts and flash floods in 2018 have affected >350 000 people by damaging agricultural land and leaving residents with insufficient nutritional sources.^25^ According to the UNICEF, climate dangers such as heat and drought, as well as a lack of critical services such as healthcare, make Afghanistan the 15th most dangerous country in the world for children.^26^ During the harsh winter months, snow accumulation causes blockage of many roads, thus making transportation of essential goods relatively difficult. Hence many civilians are unable to restock critical food supplies during this time.^27^

The implications of this crisis are many, with the most devastating being the untimely deaths of millions of children due to this worsening situation. For the few malnourished children who survive, their economic productivity is severely impaired into adulthood.^19^

## Efforts and recommendations

With the assistance of the United Nations and other humanitarian organizations, the Afghan government introduced a series of nutritional programs and agendas that centred around reconstructing the healthcare system, expanding access to primary healthcare services, increasing access to nutritious dietary commodities and creating stronger nutritional policies and regulatory bodies.^22^ Several nutrition-related policies consist of strategies on prevention and control of vitamin and mineral deficiencies.^22^

Efforts taken by organizations such as UNICEF to educate the local population include launching of awareness campaigns in Afghanistan.^22^ This allows for health and nutritional mobile services to be readily accessible and aids in increasing knowledge and awareness so that families are better able to incorporate healthy eating habits and reduce the incidence of malnourishment.^16,22^ Moreover, Afghanistan took a stand to reduce malnourishment by joining the Scaling Up Nutrition movement in 2017.^28^ This movement has implemented a permanent multisector food security and nutrition coordinating body that monitors the quality of food, implements food security measures and improves agriculture production in Afghanistan.^29^ With this service, the number of children receiving treatment for severe acute malnutrition significantly increased in 2018.^29^ Similarly, the sentinel site–based National Nutrition Surveillance System was implemented in collaboration with the Basic Package of Health Services and Essential Package of Hospital Services.^30^ These help to obtain accurate information on the number of malnourished mothers and children so that effective prevention and nutritional plans can be implemented.^30^ However, there are no data available to gauge the effectiveness of these initiatives.

Moreover, Afghanistan's Ministry of Public Health developed a pictorial community-based growth monitoring and promotion scheme to help educate illiterate Afghani women on ways to improve their diet.^31^ As a result of this initiative, a significant improvement in the nutritional status of children was noted.^31^ Also, UNICEF provides funding for the Baby-Friendly Hospital Initiative, a program that offers aid for lactating mothers admitted in hospitals or birthing centres and educates mothers on the importance of breastfeeding.^32^ There was a steep increase in the implementation rates from 2004 to 2016, indicating the potential for further success of this program.^32^

UNICEF also launched its largest-ever single-country appeal for Afghanis, which seeks $2 billion for national welfare.^20^ On the ground, UNICEF has paid the salaries of 10 000 healthcare workers as of November 2021.^20^ The organization has also supplied vital heating materials to combat the harsh winters and provided medical supplies to >1000 health facilities.^20^

In December 2021, Pakistan began sending thousands of metric tons of wheat to Afghanistan along with pledging almost $28 million worth of assistance, which includes 50 000 metric tons of wheat.^33^ In addition, the Pak-Afghan Cooperation Forum sent 353 tons of winter supplies, hygiene packs and food items in February 2022.^34^

Child malnourishment is a multivariable problem and requires various solutions from multiple angles to address the issue effectively. First and foremost, the freezing of global aid must be revived and international sanctions must be removed to provide immediate relief to the population. Second, most girls are married off before the age of 18 y and are less likely to continue higher education after marriage.^8^ This increases their chances of suffering from pregnancy and paediatric complications, including malnourishment, in the future due to a lack of adequate education. International organizations like the World Health Organization can collaborate with local authorities to ensure that strict measures are implemented to support vulnerable families so that young girls are not seen as a financial burden. Similarly, religious leaders can cooperate with local authorities to help educate the local population on complications that can arise with child marriage.

It is imperative to consider that without a qualified nutrition workforce and collaborations with institutions regarding the endorsement of policies at a local level, meeting the country’s nutrition program goals is impossible.^22^

Therefore, gaining humanitarian organization support and resuming funds from Western countries is essential to strengthen the struggling healthcare system and economic status of Afghanistan. Along with providing essential nutritional and medical support, organizations should also donate supplies such as snowploughs to help combat the brutal winter snow. Families will be better able to stock up on sufficient food supplies during the winter months, which will result in decreased rates of malnourishment and disease. International organizations can also donate medical supplies such as medications, personal protective equipment and other commodities, as well as food supplies, to alleviate the current unfolding crisis.

Additionally, to promote self-reliance in the population, global organizations can educate local leadership about farming, creating a sustainable impact within local communities.

To reiterate, the worsening situation on the ground for Afghani children requires the implementation of effective strategies to control this increasing malnutrition crisis before the country enters a state of famine. Our article highlights the predominant challenges in eradicating malnutrition and provides recommendations to overcome it. We have also included an analysis of initiatives that have proven to be beneficial for this situation. The next immediate step is to resume humanitarian aid and to remove international sanctions to avoid future complications for the vulnerable child population.

## Conclusions

Halting humanitarian aid and implementing sanctions has pushed the already food-insecure Afghani population into a starvation crisis with >3.3 million children unable to afford basic food resources, leading to dozens of deaths every week. Immediate continuation of global aid and the removal of international sanctions is crucial to alleviate this worsening crisis before the situation passes the point of no return. International and local governments must initiate nutritional awareness campaigns in the population so that they can make educated nutritional decisions. Lastly, humanitarian organizations must participate in donating valuable medical, agricultural and nutritional supplies in order to save the lives of Afghani children.

## Data Availability

All data are available in the article.
